# Emotional sounds of crowds: spectrogram-based analysis using deep learning

**DOI:** 10.1007/s11042-020-09428-x

**Published:** 2020-08-17

**Authors:** Valentina Franzoni, Giulio Biondi, Alfredo Milani

**Affiliations:** 1 Department of Mathematics and Computer Science, University of Perugia, Perugia, Italy; 2 Department of Mathematics and Computer Science, University of Florence, Florence, Italy

**Keywords:** Emotion recognition, Image recognition, Crowd computing, CNN, Transfer learning, Crowd emotions

## Abstract

Crowds express emotions as a collective individual, which is evident from the sounds that a crowd produces in particular events, e.g., collective booing, laughing or cheering in sports matches, movies, theaters, concerts, political demonstrations, and riots. A critical question concerning the innovative concept of *crowd emotions* is whether the emotional content of crowd sounds can be characterized by frequency-amplitude features, using analysis techniques similar to those applied on individual voices, where deep learning classification is applied to spectrogram images derived by sound transformations. In this work, we present a technique based on the generation of sound spectrograms from fragments of fixed length, extracted from original audio clips recorded in high-attendance events, where the crowd acts as a collective individual. Transfer learning techniques are used on a convolutional neural network, pre-trained on low-level features using the well-known ImageNet extensive dataset of visual knowledge. The original sound clips are filtered and normalized in amplitude for a correct spectrogram generation, on which we fine-tune the domain-specific features. Experiments held on the finally trained Convolutional Neural Network show promising performances of the proposed model to classify the emotions of the crowd.

## Introduction and previous work

For long time research on *sound emotion recognition* has mainly focused on the individual dimension aiming at detecting emotions either perceived by single listeners, typically through music [17] or produced by single speakers speech [8, 16, 27, 34] and expressed by fine-tuning different shades of vocal features [21, 25]. Recently, [29] introduced the innovative proposal to investigate the *emotions embedded in the crowd sounds*, collectively produced by the participants to mass events.

It is well known how a stadium of football fans can loudly express Approval or disapproval, highlighting different phases of the game, e.g., showing happiness for a goal or delusion for a missed one. In public events (e.g., concerts, receptions, parties, political meetings, protests, riots) and the public areas holding social activities (e.g., an open-air marketplace, a shopping mall, a restaurant, an airport hall), the crowd can collectively express its emotions by laughing, cheering, booing, shouting in protest, or showing a *neutral emotion*, like, for example, the background sound produced by a group quietly chatting at a party, or by a sports stadium crowd during a boring part of the match.

The innovative concept of *crowd sound emotion* is of central importance for user-oriented applications needing to understand the emotional context which influences perceptions and decisions of the individual users. It is worth noticing that *crowd sound* has its peculiarities, which demand specific management. Consider, for instance, an individual panicking in a Covid-19 social-distanced crowd, triggering a panicking crowd. The same individual in a crowd-neutral context will require different management. The expression “*the crowd roar*” [20] captures the essence of the concept of the collective emotion expressed through sound by the *collective individual*, i.e., the crowd, dynamically influencing the behavior of the *single individuals*.

Crowd sound is not the result of the simple summation of individuals’ speeches: other sounds than human speech are present, e.g., screams, whistles, hisses, claps, bumping objects. In this situation, there is a phenomenon of *mirroring, mutual tuning and synchronization*, like in a spontaneous choir. Multiple *emotional streams* can be emitted by the crowd at the same time, e.g., in the case of different groups of football fans simultaneously screaming of happiness and delusion when a team scores; or booing in protest when a penalty is issued. It is necessary to rethink the emotional classes and their different shades: a specific *crowd sound emotional model* needs to be defined.

Crowd sound emotion elicitation can be related to other forms of collective behavior analysis, such as detection of sentiment polarization [9, 10, 22] and emotional trends in social media [1, 5, 32, 33] although it presents crucial specificities. A relevant difference is that emotions in social media are filtered by conscious knowledge because they are mainly transferred and extracted from text, i.e., emotional words [36]; on the other hand, the generation of *crowd sound* requires individuals to create it collectively and simultaneously, in a massive coincidence of time and place. The individual contribution to the *crowd sound* is usually not made up of verbal utterance, but, more often, it consists of sound emissions (e.g., hisses, boos, modulated screams). Those individual sounds are less mediated by the individual cognitive knowledge level, therefore they are more connected with the psychophysical aspect of emotions. In other words, *crowd sounds* genuinely represent the collective individual and naturally embed and carry emotional content.

In this work, we extend and improve an introductive visionary study on the recognition of emotional crowd sounds in mass events, presented in the workshop SAT at the 2019 System, Men and Cybernetics IEEE Conference [17]. Rethinking the emotional classes for crowd context, we present an extension of the preliminary ideas on *crowd sound* and a *crowd-sound emotion model* implementation, using deep learning and transfer learning techniques. The resulting *crowd sound emotion recognition system* has successfully experimented on a larger dataset of real crowd sounds purposely collected. An improved set of auditory scales for the spectrogram transformation and their application to this specific domain have been experimented and assessed.

The key points of the proposed system are the sound-to-spectrogram transformation and the spectrogram image classification based on transfer learning. Since we assume that information about the emotional content of crowd sounds relies on frequency-amplitude features, the idea is to transform the labelled *crowd sound* in a set of spectrogram images used for deep learning classification training.

*Transfer Learning* (TL) techniques are applied to a Convolutional Neural Network (CNN), pre-trained on the extensive database of visual knowledge ImageNet [7], to avoid the large number of sound crowd examples needed to train a deep network from scratch. A sliding window is moved, over each original sound clip [29], in order to generate a set of spectrogram images. The images feed the Convolutional Neural Network AlexNet [23], pre-trained on ImageNet [7] and modified in the last levels in order to embed the classes of the specific crowd sound emotion model. The new levels weights are adjusted by a supervised domain-specific fine-tuning phase [2–4, 19, 31]. It is worth noticing that TL methodology is not new to sound recognition [30, 35], but its application to emotional speech is due to recent works [25]. To the best of our knowledge, this work and the preliminary [17] represent the first application to *crowd sound emotion* recognition. An improved set of auditory scales for the spectrogram transformation compared to those in [17] has experimented, and their performance evaluated.

In the following Section 2, the system architecture workflow, and the applied methodologies based on CNN, TL, and sound-to-spectrogram transformations are presented, in Section 3 experiments are described and results reported and discussed, conclusions are finally drawn in section 4.

## The system architecture workflow

**Fig. 1:**
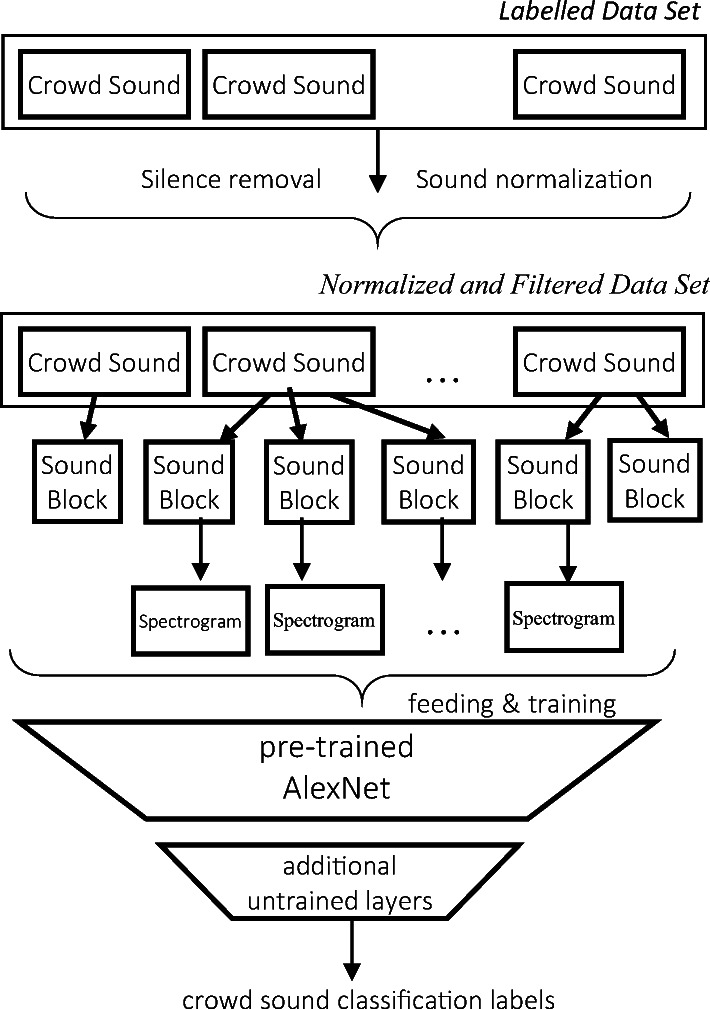
System architecture of the experimental method.

In the proposed system (see Fig. 1*System Architecture*), the organization of the information flow for the *Heterogeneous Transfer Learning (HTL)* includes two main phases:

- *sound-to-spectrograms transformation;*
- *Knowledge Transfer training.*

In the *sound-to-spectrograms transformation* phase, the sound parameters of labeled clips of varying duration are first normalized; the clips are then divided into fixed-length blocks, each of which produces, in turn, a spectrogram, labeled with the emotion from the original clip.

The* Knowledge Transfer training* phase consists first in modifying the last layers of the original CNN, according to state-of-the-art techniques [6], resetting and adapting them to the classes of the new domain, the modified CNN is re-trained using the information encoded in the spectrograms to recognize the emotional crowd labels. The fine-tuned CNN, obtained by re-training, will be used to test emotional crowd sounds recognition.

### Sound normalization

Each original sound clip has been sliced in sound blocks of *t*_*b*_ = *1* *s* using a time sliding window with *t*_*s*_ *= 0.25* *s* slide and *t*_*b*_*–t*_*s*_ = *0.75* *s* overlap. This procedure allows for obtaining images of uniform size. The sound blocks time length has been chosen experimentally, aiming to obtain a balance between accuracy in tracking frequency peak variations and reducing the computational load.

Differently from other works on speech recognition, which mainly analyze the human voice frequencies, i.e.,* 20–8000 Hz*, in this work we purposely chose to keep the whole human hearing frequency spectrum, i.e., the *20–20,000 Hz* range.

The reason for choosing this frequency range is that, although the main contribution in terms of information comes from frequencies in the voice spectrum, crowd speech samples often include additional sounds. For instance, the sound of people cheering may contain glasses shaking or hands clapping. In contrast, a booing crowd sound clip can consist of attentive sounds, clattering, movement noise, and chattering, where each sound information may prove crucial for the ultimate labeling process. Trigger reactions can be different in different cultures.

We normalized the loudness of the dataset to *−23 Loudness Units* (LU or LUFS, referenced to Full Scale), following the *EBU R128* standard [11].

### Generation of crowd-speech frequency/amplitude spectrograms

The generation of spectrogram images of single sound blocks makes necessary to choose a frequency scale in order to produce consistent homogeneous image representations. Such choice can potentially affect the analysis results because different scales emphasize different frequency ranges, thus different components of the sounds in a crowd sound block tend to emerge with different scales. In this work, we systematically analyze four frequency scales, which have been chosen for their intrinsic characteristics, expressing the different contribution in increasingly lower frequency ranges:

1. *Mel* [34], for the 4–6 kHz range
2. *Erb* [28], for the 2–4 kHz range
3. *Bark* [37], for the 0–3.5 kHz range
4. *Log* [25], for the 0.02-2 kHz range

**Fig. 2:**
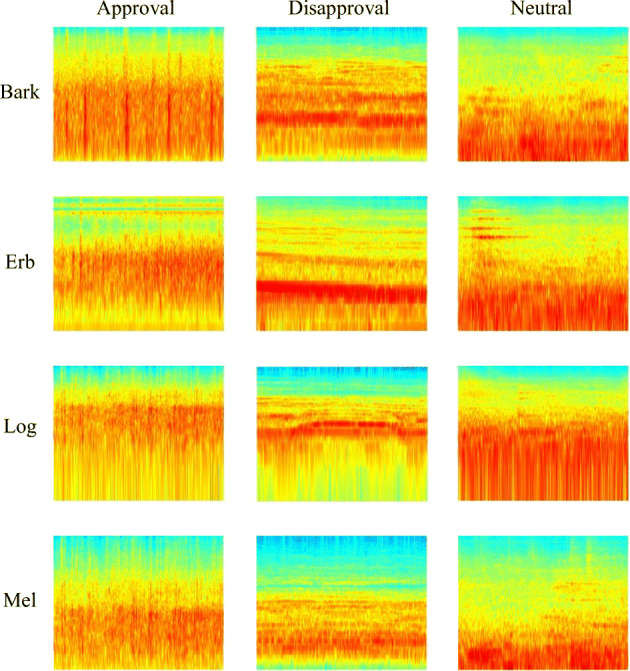
Spectrograms examples for blocks of different categories for each scale.

**Fig. 3:**
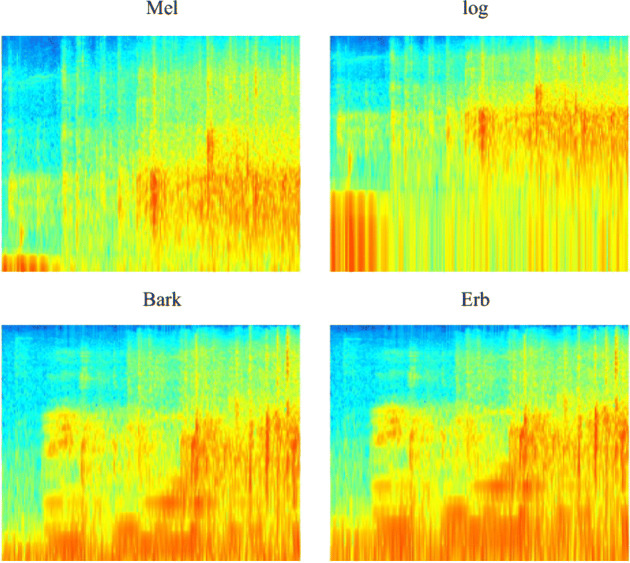
Spectrograms examples for the same block with different scales.

Each sound block computes spectrograms in all the four frequency scales: in Fig. 2, the spectrograms of a random sample per each category and scale are shown, while an example of the spectrograms for the same *1-s* segment can be seen in Fig. 3; in both figures, the x-axis represents *time*, the y-axis *frequency*, and the color intensity represents the *amplitude* of the signal.

In particular, the most widely used frequency auditory scale in the literature, related to spectrogram-based image deep-learning classification, is the *Mel* (Melodic) perceptual scale of pitches. Spectrogram-based image classification is used in the state-of-the-art for human speech and music classification. The *Mel* scale represents the sound pitch based on listener perception. A perceptual pitch of *1000 Mel* is assigned to a tone of *1000* *Hz*, *40* *dB* above the listener’s threshold. The *Mel* spectrogram represents the short-term power spectrum of a sound, transforming the input raw sound sequence in a bidimensional feature map where the *x*-axis represents *time*, the *y*-axis represents the *frequency* (*Log10* scale), and the values represent *amplitude*.

The magnitude of the generated spectrograms is of a size *257 × 259* for frequency and time, using the *jet* colormap of *64* colors, which is particularly suitable for our recognition goal, because of the luminance of colors, which is not easily garbled. On the other side, the intense luminance may generate ripples causing the recognition of non-existing pitches in the classification step [26]. This side effect has been avoided using a *hamming window*, described in the following paragraphs, which helps to smooth the discontinuities in the original non-integer number of periods in the signal.

The spectrogram images have been downsized to *227 × 227* *pixels*, which are the input dimensions for our CNN.

The *hamming-window* size is *400* samples, with a frame increment of *4.5 milliseconds*.

### Domain-specific training of the AlexNet CNN

In the experiments, the analyzed emotions of *Approval*/*Disapproval* are compared with a *Neutral* emotion, in direct correspondence with the crowd emotions obtained from clips containing people cheering/clapping, booing, and noisy background chattering in crowded events.

Visual transfer learning is employed to benefit from both the vast, existing visual knowledge base and the fast training time required. Convolutional Neural Networks trained with extensive image datasets, such as the popular *ImageNet*, have been proved to embed excellent recognition abilities [13, 18, 24]. *AlexNet,* trained on ImageNet, is one of the first of such CNN and is still among the most widely used for deep learning-based image classification.

The basic idea for transfer learning in this domain is that different layers in deep neural networks recognize various characteristics of the training domain. More precisely, the first layers in a network know low-level image features. The deeper we go in the network, the more the following layers express an increasing complexity in the recognition ability. The last layers, which implement the actual classification, reassemble with appropriate weights the previously detected image patterns in such a way that reflect the characteristics of a specific domain. In contrast to the specificity of the last layers, the basic knowledge, contained in the first layers, is common and can be shared between different domains.

Such a feature is perfectly adaptable to our domain, where the low-level features of images, e.g., edges, corners, shapes, and color distribution, are common to all the image instances in a spectrogram environment and are shared with almost any other item in the ImageNet dataset. When we consider low-level features regardless if they come from photos or geometrical images and drawings, the essential elements are common.

The Knowledge Transfer of the low-level features from the AlexNet Convolutional Neural Network is entirely feasible and fine to this aim.

AlexNet is pre-trained on a vast number of images of different categories of the ImageNet database. The learning transfer consists of modifying both its topology and weights, where the final layers are replaced with new layers specific to the crowd emotional task, i.e., fully-connected layers.

The whole network is then fine-tuned, focusing on the newly added layers, including training on the high-level features of the specific problem, i.e., the crowd sound spectrogram frequency and amplitude visual features, related to the emotion tagged in training set on the chosen scale. The global learning rate is set to a low value, to avoid modifying too much the previous original layers. A learning rate multiplier is employed to speed up the learning process of the new final layers. Transfer Learning allows for faster training time, even by several orders of magnitude, because basic image features do not need to be learned again by the network. In this way, we also obtain to require much less training samples to achieve consistent performances, compared to learning from scratch an entirely new network.

### Crowd sound dataset

The dataset used for this work has been collected by the authors from selected audio clips of real crowd high-attendance events. Different events have been chosen so that the whole dataset is composed of several sub-sets related to various events. The original audio clips have been preprocessed, as explained in sections 2.A and 2.B and labeled according to the audio content and description. Three categories are considered, namely *Approval*, *Disapproval*, and *Neutral*.

Many audio sources available both in the Web and in scientific research datasets include fake library sounds, e.g., for audio dubbing, or sounds from professional or naive acting, cannot guarantee the same authenticity and spontaneity. Authenticity is an extremely relevant feature of a corresponding dataset for emotional sounds, because, despite the type of the used deep-learning technique, the neural network can automatically extract the misleading features due to fake elements of the clip, and then be able to recognize only artificial sounds instead of original, real ones. The importance of this problem is not related only to the features that humans can easily understand in a fake sound. Thus, the authenticity of the clip should be primarily investigated and the use of any sound clip of uncertain source should be avoided.

Furthermore, audio clips recorded in real-life situations can present more complexity than purposefully studio recorded clips, e.g. uncleannessand background noise. Therefore, real-life audio clips are of higher interest to be studied for systems that should be able to recognize sounds from different environments.

The features related to a particular environment, e.g., background noises, the overall stableness of the volume or the intensity of the sound, and specific voice types, such as a crowd composed only by children, should not influence the final performance of the deep-learning classification system. For this purpose, the audio has been chosen from different situations, and fragments from the same audio clip are never used both in training and in testing phases of the classification. The various clips have been selected to share several similar characteristics (e.g., noise, continuous or rhythmic sounds) to avoid any bias introduced by considering inherently different categories.

The dataset is composed of 69 clips, each of which is split into several *1-s* blocks. The last block of each clip may be less than 1 s, still kept in the dataset. The crowd sound dataset is composed of 9515 blocks in total from 69 original clips for the three categories. Blocks containing silence segments were removed, as they were likely to affect performance, shifting the amplitude scale towards the bottom and therefore squeezing the spectrum area containing emotional information. The original number of clips, the total original duration per category, and the total number of blocks after preprocessing are shown in Table 1.

**Table 1:** Per-class clips number, blocks number and duration in the dataset

Class	Different Clips	Total duration (s)	Total of blocks
Approval	39	518	1787
Disapproval	15	118	388
Neutral	15	1874	7340
Total	69	2510	9515

### Experimental setup

A *majority-vote validation* scheme has been adopted for the experiments. Given a sound clip, a set of overlapping blocks and the corresponding spectrograms are generated, then for each spectrogram, an emotion label is predicted. In the *majority-vote validation* the class of the clip is determined by collecting the labels of all the blocks spectrograms belonging to such a clip and selecting the most represented label.

We assume that some samples, especially those corresponding to transition phases (if any) between *neutral* and *non-neutral* emotional content, maybe incorrectly labeled. However, the predominant content of the clip can still be determined by selecting the most represented label.

The first approach follows the standard practices in image classification, as used in state-of-the-art works on speech emotion recognition [25], where a dataset is partitioned in two subsets by randomly picking images and assigning them to the *training set* and *test set*, according to a given proportion. In the used training/test set ratio, 80% of images are assigned to the training set and 20% to the test set. It can be argued that such an approach, in this specific case, could easily lead to overfitting, because different spectrogram images, for example, one from the training set and one in the test set could pertain to the same clip and the subsequent derived blocks. In this case, a *bia*s is introduced indirectly providing information on the *test set* while training the neural network.

As described in section A, spectrograms are created from partially overlapping blocks of sounds extracted from clips: a random split will distribute the spectrograms between training and test set, separating blocks generated from contiguous frames which share the same information. If adjacent blocks can be remarkably similar, also non-contiguous blocks can embed very similar environmental-based information, as explained in detail in section 2.D, where the approach to select clips for the dataset is described. Therefore, with identical data in the training and test sets, the network would be able to exploit such highly similar information in the two sets, which likely leads to a lack of generalization capabilities and overfitting on the specific features of a clip.

For this reason, a second approach for a fair construction or the training/test set has been finally adopted, it consists in distributing all the blocks of different original sound clip files either in the training set or in the test set with no overlapping, to test the model recognition abilities, never-seen-before data.

In this case, the selection criterion was to maintain percentages as similar as possible to the first experiment, with the additional constraint of moving all the blocks of a specific file either to the training or to the test set. Therefore, for each category, a percentage between 80% and 90% of the data has been reserved as a training set. The adopted metric is the accuracy, calculated on the test set [15]. Five networks were fine-tuned for each experiment and each scale, and their results averaged, for a total of 40 networks and eight averaged results. All the networks were trained on an NVIDIA GeForce GTX 1070 GPU for four epochs, with mini-batches of *32* images. Both the initial learning rate and the L2 regularization factor set to 1*10^*−4*^.

## Experimental results

Although the system shows high accuracy scores for both the experimental settings, significant differences can be observed in the two cases. In particular, the second setting shows, as expected, slightly lower performance than the first one. This variation could be expected due to the reasons discussed in paragraph II.E, being it a more realistic and fair test environment. In both cases, the number of training epochs proves to be approximately the same. Although the maximum amount of training epochs was set to *4*, on average, the system reached peak performance after *1* for the first scenario and *2* for the second, with no substantial later improvement.

Results are reported in Tables 2 and 3.

**Table 2:** Results for experimental setting 1

Scale	Accuracy (Avg. over 5 re-training)
Mel	0.9983
Erb	0.9981
Bark	0.9983
Log	0.9968

**Table 3:** Results for experimental setting 2

Scale	Accuracy (Avg. over 5 re-training)
Mel	0.9292
Erb	0.9636
Bark	0.9646
Log	0.9924

No distinguishable difference can be found between the four scales in the first experimental setting (see Table 2), probably due to the overfitting issue mentioned in section 3.E.

In the second experimental setting (see Table 3), which is more realistic, i.e., not overfitting, the average accuracy scores calculated on the single blocks’ labels are still above *90%* on average in all the experiments. Some differences can be observed in terms of performance between the considered scales. In particular, *Bark* and *Log* scales perform better than *Erb* and *Mel*, suggesting that the relevant features of the crowd-sound domain are located in the lower part of the frequency spectrum. Table 4 shows the confusion matrix for the third network trained with spectrograms generated by the *Mel* scale. The vast majority of classification errors occur between the *Neutral* and *Approval* categories, with samples of *Neutral* attributed to *Approval*. Such a pattern suggests that the *Disapproval* class holds more distinguishable characteristics than the other two.

**Table 4:** Confusion matrix for Mel scale on each spectrogram of network 2

Real/Predicted	Approval	Disapproval	Neutral
Approval	229	6	0
Disapproval	0	51	0
Neutral	119	34	883

We recall that in Table 4 is represented only one of the *20* networks trained in the five scales for the second experimental setup, as a sample. The values in the table are thus related to every single vote, i.e., each classification attempt, on which we choose the most represented class for each sound clip. The accuracy scores show a similar result for all the five re-trained networks of the majority-vote classification scheme explained in section 2.E (see Table 5), where the *Log* and *Mel* scales show a perfect classification. We also notice that for the *Bark* scale, all but one of the re-trained networks misclassify two disapproval class samples as *Approval*, while for the *Erb* scale, the same misclassification happens only once (on the same file misclassified by *Bark*).

**Table 5:** Results for the majority-vote classification scheme

Scale	Network	Accuracy	Correct classification (majority classification)	Wrong classification (majority classification)
Mel	0	0.8994	12	0
Mel	1	0.9561	12	0
Mel	2	0.8797	12	0
Mel	3	0.9781	12	0
Mel	4	0.9327	12	0
Erb	0	0.8805	12	0
Erb	1	0.9728	10	2
Erb	2	0.9849	12	0
Erb	3	0.9917	12	0
Erb	4	0.9879	12	0
Bark	0	0.9773	10	2
Bark	1	0.9433	10	2
Bark	2	0.9652	12	0
Bark	3	0.9758	10	2
Bark	4	0.9614	10	2
Log	0	0.9947	12	0
Log	1	0.9894	12	0
Log	2	0.9992	12	0
Log	3	0.9803	12	0
Log	4	0.9985	12	0

The behavior on the overall results suggests that the *Log* scale guarantees the best results for the proposed classification task, both in terms of single spectrograms and whole files. The *Erb* scale obtained better results than the others, where the retrained networks misclassify two *Disapproval* class samples as *Approval* and, since the misclassification happens on the same files, a further study could investigate their particular features to understand in which real cases the classification may fail.

Since, to the best of our knowledge, this work represents the first attempt to use a spectrogram-based approach with crowd emotional sounds, there are no datasets or results in the literature to compare our results. To give a general idea on the algorithm performance, we can provide a coarse-grained comparison with the same approach applied to individual-speech emotional sound [25], aware that the specific features of the two cases are not strictly comparable. On crowd sounds, the performances are improved on average of *10%*. Another element why such comparison is only for intuition is that the average results are given for all the emotional classes (i.e., experimented emotional model), which differ from individual to crowd emotions.

## Conclusions and future developments

The main contribution of this work is to introduce a model for crowd sound emotions and to show the feasibility of the implementation of crowd sound emotions recognition system with spectrogram-based techniques integrated with CNN convolutional neural networks. Since the presented implementation and dataset is to date the first one on crowd sound emotions, we have to point out that our experiments cannot be directly compared to any previous study in literature. A general comparison has been discussed, by comparing the experimental results for crowd sound emotion with the result available for a similar algorithm on a different emotional model, i.e. on the domain of individual-speech emotions studied in [25]. Note that our domain and emotional model show a 10% improvement with respect to the 80% average accuracy of the individual-speech domain on every scale, even in the second experimental setting where we lower the performance gaining a better consistency. The results support the conclusion that the transfer learning AlexNet-CNN spectrogram-based approach is suitable for the crowd emotional sound domain. The results presented in this work also prove that it is possible to develop a real-time emotional crowd sound recognizer for the given categories. Potential application fields of such a classifier range from user context-based interfaces to safety and emergency domains in crowd context.

Priority in future research will include a thorough analysis of the properties to model emotional crowd sounds to deeper understand and characterize the distinctive traits of each *crowd sound emotion* class. While it is relatively easy to recognize the positive/negative polarity of crowd sound emotions, there are still open questions about the type of emotion classes. *Are there basic emotions that specifically characterize crowd sounds? Are individual-based emotional models (*e.g.*, Ekman *[12]*) adequate for the collective individual expressing through crowd sound? Can a crowd sound show surprise, embarrassment, sadness?*

Another issue is the complexity of *crowd sound patterns*. In the presented experiments, we have considered *short-term crowd sounds*. On the other hand, it would certainly be worth considering longer intervals of time and the dynamic evolution of *crowd sound patterns*. A typical example is the pattern of growing crowd excitement followed by a joy burst like those associated with game actions. This patterns should be considered as an emotional unit, instead of merely focusing on short-term separated sound blocks.

An aspect worth investigating is how to model and distinguish the *different emotions streams* generated by different crowd subsets, e.g., sounds associated with a goal/no goal situation in football matches, where the supporters of opposing teams would simultaneously produce different sounds, sometimes opposite in term of the conveyed emotion. In this regard, from the emotional model point of view, it is also necessary to understand if *mixed crowd sound emotion states* can be described by the compounding *pure crowd emotion* or they require to be explicitly distinguished.
